# Quantifying the Full Damage Profile of Focused Ion Beams via 4D‐STEM Precession Electron Diffraction and PSNR Metrics

**DOI:** 10.1002/smtd.202502258

**Published:** 2026-02-12

**Authors:** Mateus G. Masteghin, Zabeada P. Aslam, Andy P. Brown, Mark J. Whiting, Steven K. Clowes, Roger P. Webb, David C. Cox

**Affiliations:** ^1^ Advanced Technology Institute University of Surrey Guildford UK; ^2^ DTU Nanolab Technical University of Denmark Kongens Lyngby Denmark; ^3^ School of Chemical and Process Engineering University of Leeds Leeds UK; ^4^ School of Mechanical Engineering Sciences University of Surrey Guildford UK; ^5^ Ion Beam Centre University of Surrey Guildford UK; ^6^ National Physical Laboratory Teddington UK

**Keywords:** 4D‐STEM, damage profile, diffraction‐based characterization, focused ion beam (FIB), ion implantation

## Abstract

Focused ion beams (FIBs) are used in applications such as circuit repair, ultra‐thin lamella preparation, strain engineering, and quantum device prototyping. Although the lateral spread of the probe is overlooked, it becomes critical in precision tasks such as impurity placement in host substrates, where accurate knowledge of the ion‐matter interaction profile is essential. Existing techniques characterize only the beam core, where most ions land, thus underestimating the full extent of the point spread function (PSF). In this work, we use four‐dimensional scanning transmission electron microscopy (4D‐STEM) to resolve the ion beam tail at defect densities equivalent to <0.1 ions ${\mathrm{nm}}^{-2}$. Convergent beam electron diffraction (CBED) patterns were collected in calibration regions with known ion fluence and compared to patterns acquired around static dwell spots exposed to a 30 keV ${\mathrm{Ga}}^{+}$ beam for 1–10 s. Cross‐correlation using peak signal‐to‐noise ratio (PSNR) revealed that 4D‐STEM datasets are ultra‐sensitive for defect quantification and more robust against scanning artifacts than conventional dark‐field imaging. By extending beyond conventional core‐focused resolution metrics, this approach enables comprehensive mapping of ion‐induced damage—notably at ultra‐low doses—providing a more accurate picture of FIB performance for application‐specific optimization.

## Graphical abstract

Four‐dimensional scanning transmission electron microscopy (4D‐STEM) is used to acquire multiple convergent beam electron diffraction (CBED) patterns around focused ion beam (FIB) dwelled spots. Peak signal‐to‐noise ratio (PSNR) analysis, following calibration with well‐known implantation dose squares, enables full quantification of the FIB profile, including its low‐dose tails.

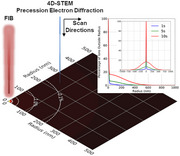

## 1 Introduction

Focused ion beam (FIB) systems are indispensable tools in nanofabrication, offering high spatial resolution for ion implantation [1, 2, 3], milling [4], and other material modifications such as strain engineering [5] or composite deposition [6]. In advanced applications such as quantum device fabrication, where atomic‐scale precision is paramount [7], understanding the full spatial extent of ion‐induced damage becomes crucial. This is particularly important in the low‐dose regime, where even very limited ion exposure can degrade the coherence properties of quantum bits (qubits) [8, 9] or alter the optical properties of quantum dots [10]. Accurate characterization of ion beam damage profiles could not only reveal intrinsic limitations of FIB techniques but also provide a foundation for refining microscope optics, developing mitigation strategies, and tailoring tool performance to meet the stringent requirements of emerging quantum technologies [3, 11].

Drezner et al. [12] discussed several techniques to measure the ion beam profile and their limitations, such as scanning across heterostructures, knife‐edge methods, and resist‐based imaging. In the heterostructure approach, the beam is scanned across a material interface (e.g., Si/SiGe), and the resulting contrast (or gray levels) is used to infer the beam shape. This approach is discussed below. While this method provides good lateral resolution, it requires dedicated samples and is sensitive to interface quality. Knife‐edge techniques involve scanning the beam across a sharp boundary (e.g., a metal edge or a patterned mask) and analyzing the resulting signal gradient. These methods are straightforward but often suffer from convolution effects and limited sensitivity to low‐dose tails. Resist‐based imaging, where ion exposure modifies a resist layer, offers high contrast but lacks quantitative accuracy and is limited by resist sensitivity thresholds. Hence, most techniques are only accurate for determining the full width at half maximum (FWHM) of the ion beam “core,” or what we will refer to here as “imaging resolution.”

To overcome these limitations, we present a novel application of four‐dimensional scanning transmission electron microscopy (4D‐STEM). We propose the use of 4D‐STEM to map ion‐beam‐induced damage with high sensitivity and nanoscale spatial resolution in the low‐dose regime. In 4D‐STEM, a convergent beam electron diffraction (CBED) pattern is recorded at each scan position, enabling detailed analysis of the local crystal structure. By comparing CBED patterns acquired in pristine regions with those obtained from ${\mathrm{Ga}}^{+}$‐implanted calibration squares (30 keV, doses from 0.09 to 5 ions ${\mathrm{nm}}^{-2}$) in a 30 nm‐thick silicon membrane, we extract peak signal‐to‐noise ratio (PSNR) values and construct a calibration curve that relates PSNR to ion dose. Using this calibration, we then scan the electron beam around holes created by static‐dwell FIB exposures in the same membrane, obtain pixel‐wise PSNR values, and interpolate these on the calibration curve to convert them into local ion densities. This method is uniquely suited to probe the beam tail: while it lacks sensitivity in the highly damaged beam core—where diffraction contrast is lost—it is exceptionally sensitive to subtle crystallinity changes in the low‐dose periphery. As such, it provides a powerful tool for quantifying the spatial extent of minimal ion‐induced damage, a capability central to deterministic ion implantation and emerging quantum‐device fabrication.

Recent advances have positioned 4D‐STEM as a powerful and increasingly popular technique for assessing crystal quality. It has been used to map strain [13], identify defects [14], and reconstruct phase information with atomic resolution [15]. Its ability to capture rich diffraction data at each scan point enables new insights into local structure and disorder, making it a valuable tool in both fundamental research and applied materials science. Our work extends the application portfolio for 4D‐STEM, including the use of PSNR‐based analysis for quantitative ion beam point spread function profiling. This approach complements existing techniques [12, 16] and provides a robust, high‐resolution method for mapping ion beam tails, an area where traditional methods often fall short.

## 2 Results and Discussion

### 2.1 Limitations of Conventional FIB Resolution Measurements

**Figure 1:**
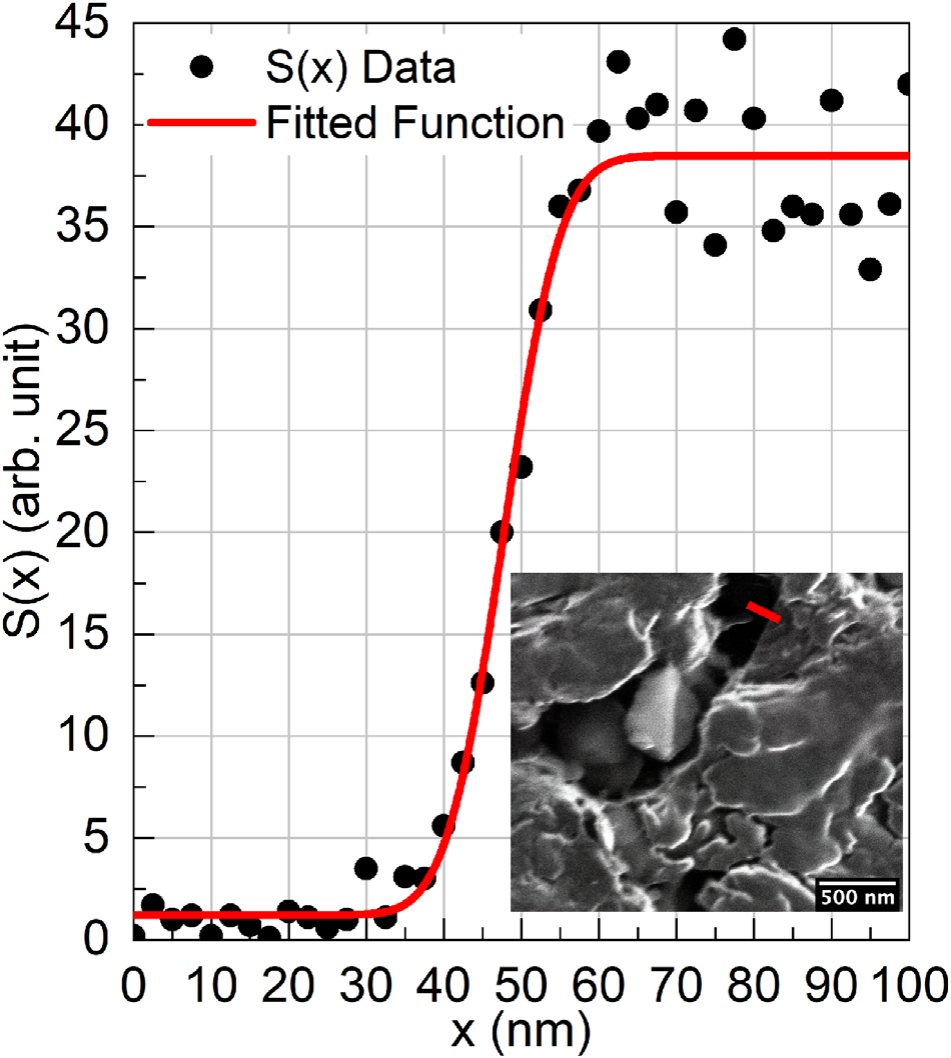
Conventional imaging resolution measurement using linear grayscale profile across a heterojunction. Inset: A 1024×943 pixel^2^ secondary electrons micrograph obtained by scanning a 30 keV ${\mathrm{Ga}}^{+}$ beam such as around 4200 electrons per pixel is used to produce the image. The red line on the top‐middle part of the inset figure represents the region where a grayscale profile S(x) was collected. The data was fitted using Equation 2 to give imaging resolution 2σ=11.9 nm or a FWHM=14.0 nm.

FIB manufacturers started adopting knife‐edge measurement as a standard to assess their system resolution. This method consists of moving either a solid material under the beam (e.g., nanomanipulator) or scanning the beam across a heterogeneous interface [17]. This technique assumes a perfectly circular beam (i.e., no stigmatism) with a Gaussian intensity distribution [18, 19]. In the case of a solid knife edge in position $x_{e}$ and a beam centered on the origin, the measured beam current is given by [20, 21]

(1) $$\begin{array}{c} I\left(x_{e}\right)=\int_{x_{e}}^{+\infty }\int_{-\infty }^{+\infty }I_{\left(x,y\right)}dxdy=\\ \int_{x_{e}}^{+\infty }\int_{-\infty }^{+\infty }\frac{I_{0}}{2\pi \sigma^{2}}e^{-\frac{x^{2}+y^{2}}{2\sigma^{2}}}dxdy=\frac{I_{0}}{2}\text{erfc}\left[\frac{x_{e}}{\sqrt{2}\sigma }\right] \end{array}$$

where $I_{0}$ is the total beam current, σ is the standard deviation of the beam width, and ***erfc*** is the complementary error function. For the case of scanning the beam across a heterogeneous interface, where the secondary electron emission is K multiplied by the secondary electron yield for each side of the interface (γ and δ), the measured grayscale S plot as a function of beam position is given by

(2) $$S\left(x\right)=K\left[\frac{\gamma +\delta }{2}+\frac{\gamma -\delta }{2}\text{erf}\left(\frac{x-x_{i}}{\sqrt{2}\sigma }\right)\right]$$

where $x_{i}$ is the position of the interface and ***erf*** is the standard error function. For both methods, the quoted imaging resolution is 2σ, which is often referred to as the “spot size” or “probe size.” An example of the latter method is shown in Figure 1, in which the inset displays a secondary electron image obtained after scanning a 30 keV, 7.5 pA ${\mathrm{Ga}}^{+}$ beam across a heterogeneous gold/carbon interface, and the grayscale profile S(x) is extracted across the region marked by the red line in the image. In this example, from fitting S(x) to Equation 2, the imaging resolution of 2σ=11.9 nm is obtained. For comparison, the corresponding Gaussian full width at half maximum (FWHM) is given by $\mathrm{FWHM}=2\sqrt{2\ln2}\;\sigma \approx 2.35\sigma$ = 14.0; however, the 2σ definition was adopted here because it corresponds directly to the $1/e^{2}$ beam radius as defined in ISO 11146‐1:2021.

A comprehensive summary of the challenges associated with using these methods is provided by Drezner et al. [12]. When a wire is imaged whilst positioned on the top of a Faraday cup, the wire roughness is typically larger than the beam radius. Conversely, when a smooth nanomanipulator is moved between the pole‐piece and the Faraday cup, the uncertainty of the piezo movement is often comparable to the beam point spread function. Therefore, in neither operational mode is the condition of a beam profile smaller than the transition regions met. In the alternative approach of scanning the beam across a heterogeneous interface, the method risks interface smothering due to material sputtering, which must be mitigated by a fast dwell time. Nonetheless, when dwelling a 7.5 pA beam for approximately 100 ns, fewer than 3 electrons per pixel are generated [22, 23], resulting in a low signal‐to‐noise ratio and likely obscuring the beam tails.

### 2.2 ADF‐STEM Imaging of FIB‐Induced Damage Around Dwelled Spots

**Figure 2:**
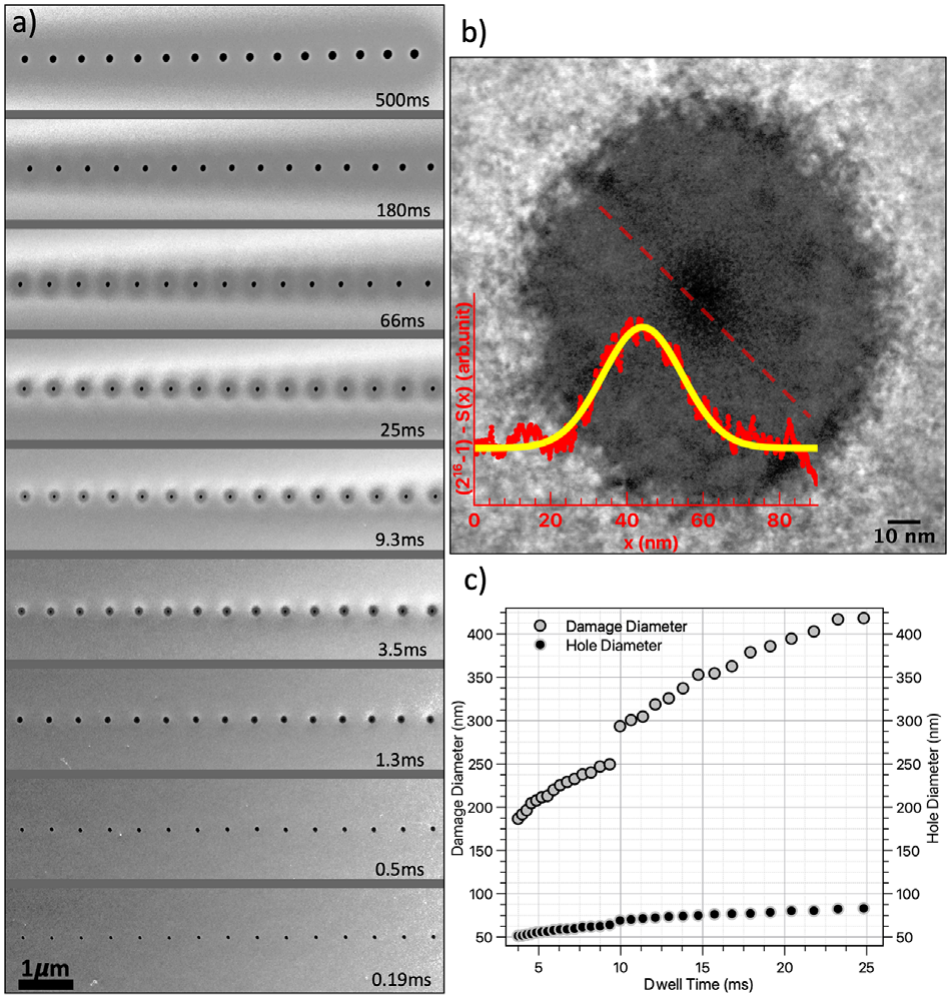
Imaging resolution versus damage profile study using annular dark‐field scanning transmission electron microscopy (ADF‐STEM). a) ADF‐STEM images around spots dwelled with a 7.5 pA 30 keV ${\mathrm{Ga}}^{+}$ beam. The times on the bottom‐right of each image is the dwell time for the far‐right spot, with exponential increments between them. Scan field of view corresponds to 8μm. b) High‐magnification high‐angle annular dark‐field (HAADF) STEM image of the initially drilled hole. The image shows a central darker region, corresponding to the hole or very thin areas, surrounded by a dark‐grey annular region, indicative of damaged (likely amorphous) areas. The untouched crystal appears in light grey. The red dashed line marks the region where the grayscale profile was obtained, which is plotted in the red inset. The yellow curve represents a Gaussian fit with a full width at half maximum equivalent to the imaging resolution obtained in Figure 1. c) Evolution of hole size and the corresponding surrounding damage, demonstrating a progressively higher damage profile relative to the hole diameter.

The challenge of observing beam tails can be demonstrated with a simple STEM experiment. A 30 keV, 7.5 pA ${\mathrm{Ga}}^{+}$ beam was dwelled in spot mode for times ranging exponentially from 0.08 to 500 ms. The 30 nm‐thick silicon single‐crystal membrane was oriented along the $\langle 001\rangle$ zone axis and imaged using the annular dark‐field (ADF) detector (Figure 2a). When the first hole was observed (≈10 ms), it was imaged at a higher magnification, and a grayscale profile across the darker central region (taken across the red dashed line) was obtained (Figure 2b). The inset in Figure 2b shows a normalized grayscale profile in arbitrary units to avoid inversion of the Gaussian shape caused by the lower intensity of the hole region in the original 16‐bit TIFF image (maximum intensity = $2^{16}-1$); the intensity corresponds to electrons scattered into the annular detector at collection angles greater than 0 mrad. A Gaussian fit of S(x) resulted in a full width at half‐maximum (FWHM) of around 15 nm. It is evident that the imaging resolution corresponds to the region where most ions arrive, leading to increased material sputtering. Substantial damage occurs in the area surrounding the dwelled pixel, as highlighted in Figure 2c, which presents plots of damage radii and hole diameter as a function of dwell times. The majority of ions concentrate near the center of the spot, leading to rapid material removal and hole formation. However, ions arrivals increasingly contribute to damage in the surrounding regions due to the broader tails of the beam profile and scattering effects such as transversal collision cascades. As a result, the damage continues to accumulate laterally as the rate of hole diameter growth slows down, leading to a steeper increase in overall damage compared to hole size. Hence, although the focused ion beam has a narrow core responsible for milling, its spatial profile includes extended reach that contributes to ion‐matter interactions. These interactions are responsible for the creation of point defects [11, 24], amorphization [25, 26], and strain generation [5]. Therefore, the ion beam community would benefit from a highly sensitive metrology framework capable of characterizing beam tail effects in nanoscale FIB patterning.

Based on the data in Figure 2, STEM imaging alone should be sufficiently sensitive to estimate the extent of the ion beam tail. However, Figure 2c reveals a discontinuity in the measured hole and damage sizes across two different micrographs acquired at different compustage positions (15 dwelled spots per ADF image, but a continuous 80μm‐long scan line with the FIB), which indicates an optical off–axis artifact. In addition, the intermodes color thresholding method cannot reliably segment both the gray damaged region and the lighter outer region in a single step, as the latter also constitutes damage but requires separate processing due to contrast inversion, introducing uncertainty. We, therefore, performed an ADF‐STEM experiment to calibrate grayscale contrast as a function of ion dose (Figure S1, Supporting Information). However, due to similar optical off–axis artifacts, a gradual offset in grayscale levels was observed across the scan (see Supporting Information). To overcome these limitations, we propose a 4D‐STEM approach that would provide a comprehensive dataset at each scan position, enabling advanced post‐processing (e.g., center‐of‐mass correction) to mitigate such artifacts.

### 2.3 Establishing a PSNR × Dose Relationship Using Defined Ion‐Dose Standards

**Figure 3:**
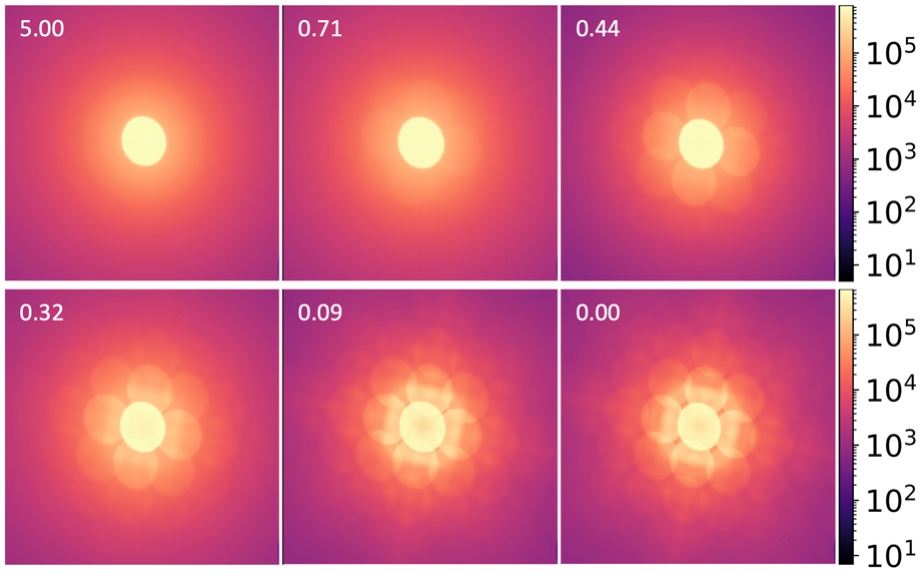
10 000 integrated CBED patterns obtained by scanning the 0.8 degrees precessed 100 keV, 12 mrad convergence, 100 pA probe current with a 1 ms dwell time per pixel. The CBED patterns have dimensions of 512×512 pixels^2^ and are plotted using a logarithmic intensity scale. The numbers at the top‐left of each CBED pattern indicate the dose (in ions ${\mathrm{nm}}^{-2}$) used in the corresponding calibration square, with 0.00 representing the pristine region.

**Figure 4:**
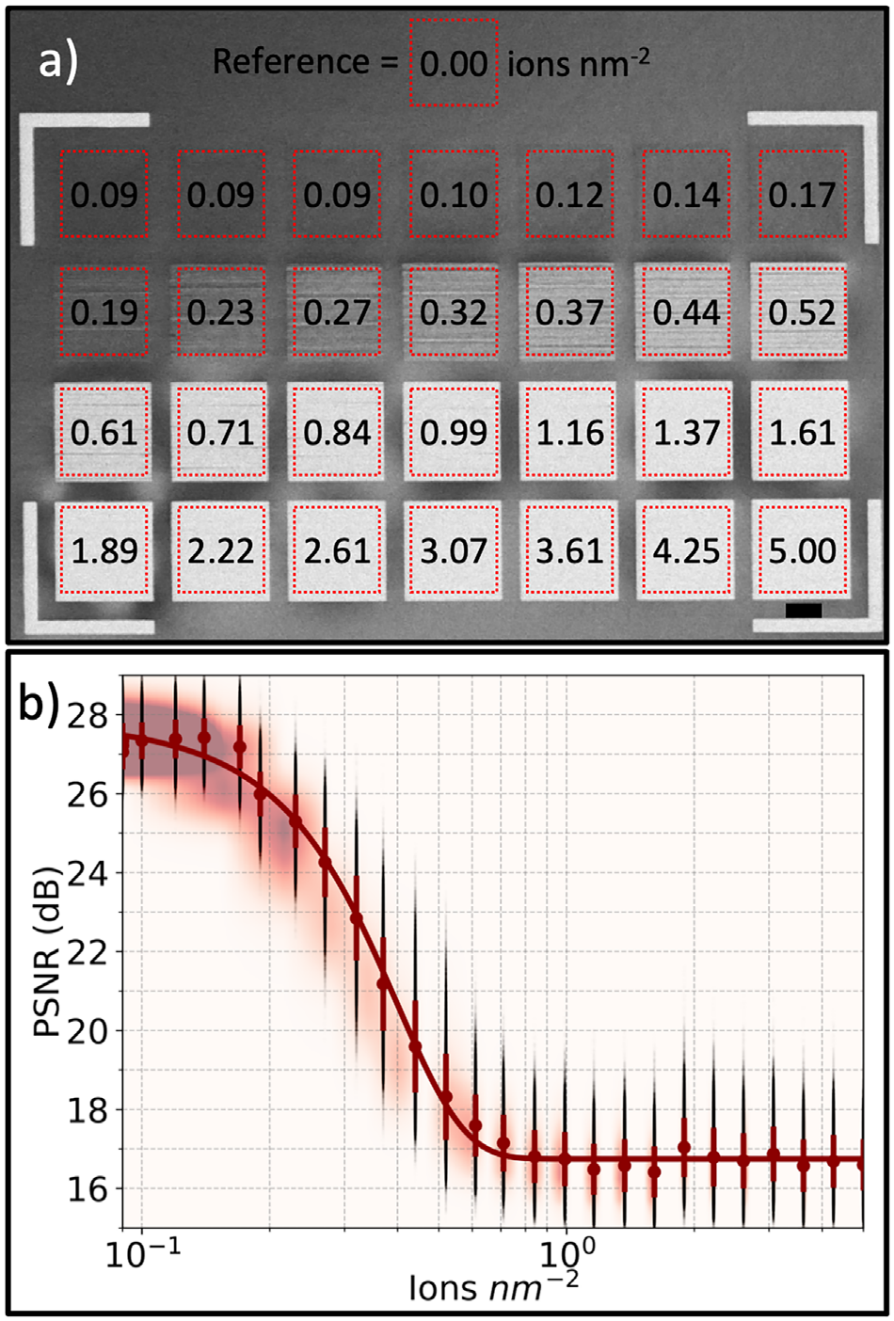
Calibration of peak signal‐to‐noise ratio (PSNR) as a function of ion dose. a) Low‐angle Annular dark‐field STEM (LAADF‐STEM) image of the calibration region, where the numbers indicate the ion implantation dose in ions ${\mathrm{nm}}^{-2}$. Red dashed squares mark the $4\;\mu m^{2}$ areas scanned to acquire the 4D‐STEM data used for PSNR analysis. b) PSNR values plotted as a function of ion dose. Black dots represent individual PSNR values (100 000 per dose) calculated from the CBED patterns in each calibration region. Red dots show the mean PSNR for each dose, with vertical bars indicating the standard deviation. Reddish blurred regions underneath the plot corresponds to heat distribution of the PSNR values for each dose. The red curve is a fit of the data using a Gaussian error function.

Figure 3 displays a series of convergent beam electron diffraction (CBED) patterns acquired using 100 keV electron precession—a tilted electron beam rotation around the optical axis producing a quasi‐kinematical diffraction [27]—from the calibration regions subjected to varying ion implantation doses. To enhance visualization, each pattern represents the integration of 10 000 diffraction patterns (DPs) collected from the corresponding calibration square shown in Figure 4a. The numerical values in the top‐left corner of each pattern indicate the ion dose in ions ${\mathrm{nm}}^{-2}$, ranging from 0.00 (pristine) to 5.00 ions ${\mathrm{nm}}^{-2}$. As the dose increases, a progressive degradation in the CBED quality is observed, culminating in complete amorphization. From those images, it is clear that CBED patterns offer a continuous grayscale intensity distribution across the entire diffraction pattern, in contrast to the binary‐like contrast of spot patterns obtained at lower convergence semi‐angles (parallel beam). To achieve this, a relatively high convergence angle was chosen to promote disk overlap and minimize regions of near‐zero intensity. This richer intensity variation enhances the sensitivity and robustness of peak signal‐to‐noise ratio (PSNR) analysis, [28, 29] enabling a highly sensitive calibration of the ion dose based on the degradation in the CBED patterns.

CBED patterns are highly sensitive to experimental conditions, alignments precision, and beam coherence; which combined with the logarithmic sensitivity of PSNR to small intensity differences, justify the lower observed values even in nearly undamaged regions. Importantly, the use of PSNR in this context is not to assess visual fidelity, as in image compression, but rather to quantify structural deviation in the reciprocal plane. In crystalline materials, CBED patterns emerge from the coherent elastic scattering of a convergent electron probe by the periodic atomic lattice, resulting in well‐defined diffraction disks that exhibit characteristic symmetry and intricate fine structures. When ion implantation occurs, it introduces lattice disorder and partial amorphization, disrupting long‐range order and enhancing diffuse scattering, which in turn redistributes intensity across the pattern. Although CBED is not strictly governed by the Bragg condition, due to the angular spread of the incident beam, the diffraction contrast remains highly sensitive to the integrity of periodic structures [30]. As a result, PSNR serves as a robust quantitative metric to detect deviations from the pristine diffraction signature. High PSNR values indicate strong similarity to the undamaged crystalline reference, suggesting minimal structural disruption, whereas lower values reflect increased deviation, consistent with greater lattice disorder.

Based on the PSNR analysis of the CBED patterns obtained from the regions marked in Figure 4a, a plot of PSNR as a function of ion dosage can be obtained and is depicted in Figure 4b with the abscissa plotted in a $\log_{10}$ scale. The PSNR dependence follows a smooth sigmoidal transition between two asymptotic states, being the regime at higher dosage consistent with the expected amorphization of the silicon single‐crystal and the lowest doses approaching a regime in which just a few more ions per $\;nm^{2}$ do not contribute to additional amorphization at the detection limit of the technique. To model the smooth transition observed in the dataset, we employed a sigmoidal fitting function based on the Gauss error function, defined as:

(3) $$f\left(x\right)=a\cdot \mathrm{erf}\left(b(x-c)\right)+d$$

where erf(x) is the standard error function, and a, b, c, and d are fitting parameters.

SUSPRE calculations [31] suggest that amorphization in silicon implanted with 30 keV ${\mathrm{Ga}}^{+}$ ions occurs at a dose of approximately 5 ions ${\mathrm{nm}}^{-2}$. However, our PSNR measurements indicate that the onset of amorphization may occur at significantly lower doses—up to five times smaller than predicted. This discrepancy arises because such calculations typically define amorphization as the point at which each ion collision displaces a host atom. In reality, crystallinity is not a binary property; long‐range order can be substantially disrupted well before complete atomic displacement occurs. A crystal cannot be considered “partially amorphous” in a straightforward way, as the periodicity of the lattice is already compromised at that stage. Reduced amorphization thresholds in thin semiconductor specimens can occur because free surfaces modify cascade recombination, promote defect clustering, and facilitate stress relaxation compared to bulk materials. Other factors may include surface melting at lower temperature, although this is less likely at the fluences used in this study. These effects can lead to amorphization onset significantly lower than those predicted by SUSPRE, which assumes an infinite bulk target. Consequently, the “amorphization onset” in SUSPRE must be interpreted with caution: it is model‐dependent, geometry–blind, and sensitive to the user's choice of critical point‐defect density, whereas in‐situ TEM defines amorphization experimentally via the disappearance of crystalline diffraction contrast. Cerva and Hobler [32] reported the crystalline‐to‐amorphous transition as the point at which a critical fraction of atomic sites is displaced—commonly taken as about 23%, corresponding to a defect density of 1.15 × 10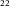 ${\mathrm{cm}}^{-3}$ for silicon. Based on our observed threshold of conservatively 1 ion ${\mathrm{nm}}^{-2}$ for a 30 nm Si membrane, SRIM [33] predicts ∼274 vacancies per ion, yielding an estimated critical point‐defect density of ∼9.1 × 10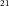 ${\mathrm{cm}}^{-3}$, or about 18.3% of the silicon crystal. These values are close to those reported by Cerva and Hobler using TEM imaging, suggesting high accuracy and, notably, increased sensitivity of our 4D‐STEM/PSNR approach, with the added advantage of requiring no additional sample preparation after ion implantation. A more nuanced understanding of this transition will be explored in a forthcoming study, combining molecular dynamics simulations [34] with the experimental 4D‐STEM measurements.

### 2.4 2D Ion Density Reconstruction Around Dwelled Spots

**Figure 5:**
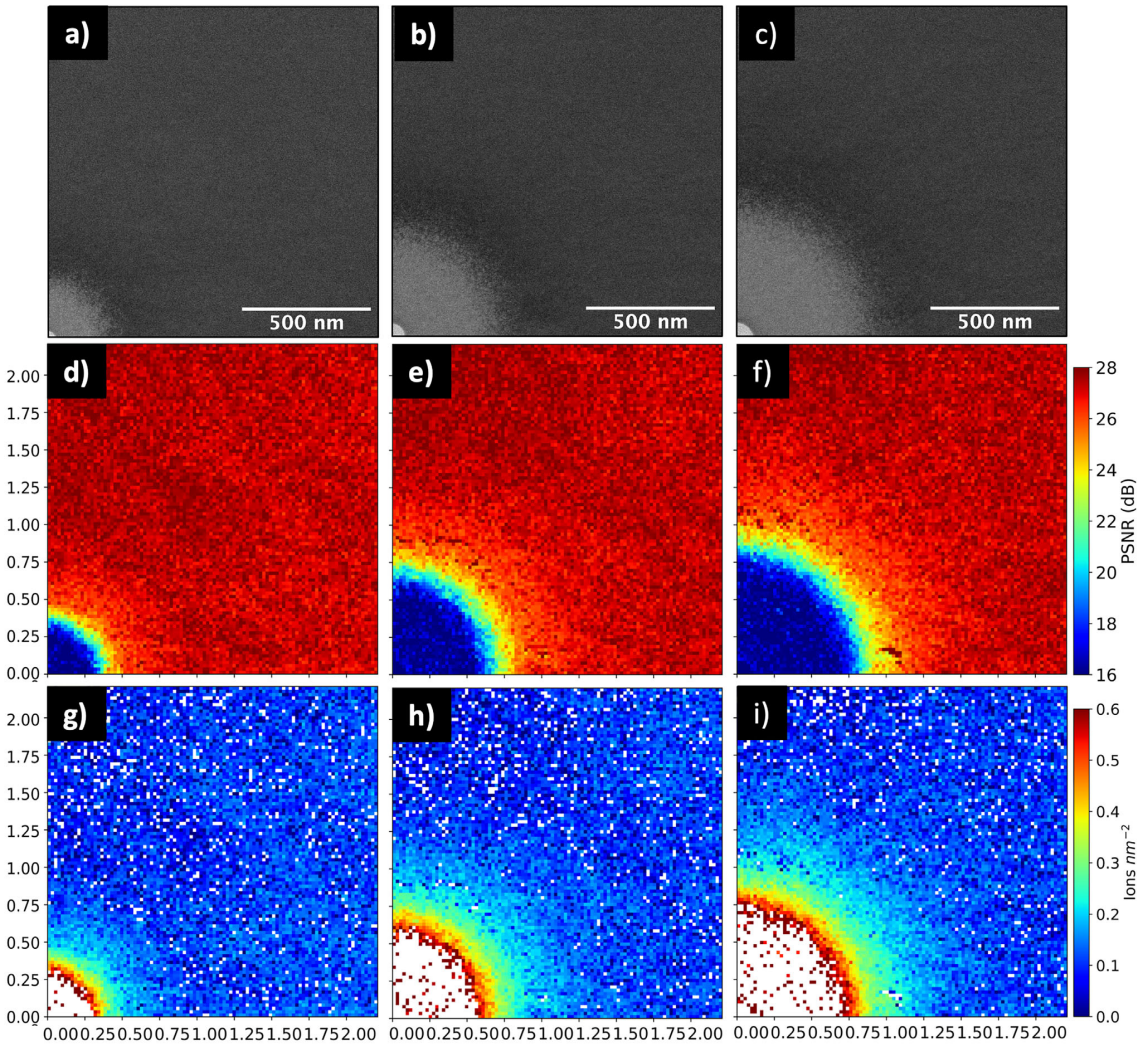
Focused ion beam‐induced damage analysis quadrants with its bottom‐left corner aligned at the center of a milled hole. a–c) ADF‐STEM images illustrating the damage across the quadrant with contrast defined by scattering events. d–f) Averaged PSNR values obtained by comparing each 100×100 scanned pixels from the 4D‐STEM dataset to 10 randomly selected CBED patterns from a pristine reference region. g–i) 2D ion dosage maps derived by converting the PSNR values to ion dose (ions ${\mathrm{nm}}^{-2}$) using parameters from the Gaussian error function fit. White regions indicate PSNR values outside the plateaus in Figure 4, corresponding to either minimal or saturated damage levels. The left, middle, and right columns correspond to dwell times of 1 s, 5 s, and 10 s, respectively.

Figure 5 displays images of a quadrant where an ion beam is held for (1 s, 5 s, and 10 s), with the beam center positioned at the bottom left in each panel. The upper three images (a–c) are low‐angle annular dark‐field (LAADF‐STEM) images, the middle set (d–f) depicts 2D PSNR maps, and the bottom trio (g–i) illustrates the calculated ion dosage surrounding the FIB‐milled hole. As previously discussed, LAADF micrographs are highly sensitive to beam‐induced damage, and it is evident that damage reaches distances of up to approximately 500 nm from the irradiation site. A quantitive measure of this damage is obtained from the PSNR images of the same field of view. Here, each CBED pattern was compared to ten randomly selected reference patterns from an undamaged region, as described in the Experimental Section, and the mean PSNR was calculated. Using the fit parameters derived from the calibration curve in Figure 4b, a 2D heat map of ion density was reconstructed, illustrating the spatial distribution of defects around the beam incidence point. It is important to note that the resulting point spread function (PSF) is sample‐specific and strongly dependent on membrane thickness, as damage arises not only from the primary beam but also from ion scattering and secondary effects within the crystal. While thinner membranes provide a closer approximation to the primary beam profile, they reduce signal strength and narrow the transition between intact and fully amorphized regions. In principle, monolayers or very thin 2D materials would offer the most accurate representation of the beam‐only profile, without significant sample‐induced contributions. White pixels in the ion distribution maps indicate regions where the PSNR falls below the baseline value, corresponding to ion doses exceeding 5 ions ${\mathrm{nm}}^{-2}$. These areas are either fully amorphized or represent locations where the 30 nm thick membrane has been perforated. Although the region away from the damage shows a small ion density (<0.1 ions ${\mathrm{nm}}^{-2}$), this highlights the high sensitivity of 4D‐STEM to subtle CBED pattern variations likely due to slight off‐axis probing considering the relatively long distance to which reference patterns were collected (tens of microns). Importantly, the uniform signal across the area confirms the absence of scanning artifacts, unlike ADF imaging over large fields of view.

To visualize the full point spread function (PSF) of the FIB‐induced damage, the PSNR maps in Figure 5d–f were symmetrically mirrored in two steps: first vertically to the left, and then horizontally downward. This process effectively reconstructs a full radial profile centered on the beam impact point, representing a complete damage distribution with a damage profile described by Equation S1 in the Supporting Information. Figure S2 in the Supporting Information shows the profiles acquired in a low‐dose regime, from 0.09 ions ${\mathrm{nm}}^{-2}$ to 5 ions ${\mathrm{nm}}^{-2}$. This contrasts significantly with the higher dose regimes used in the study by Drezner et al. [12], where TEM experiments and TRIM modelling were employed to characterize amorphization in single‐crystal silicon at doses exceeding 10–100 ions ${\mathrm{nm}}^{-2}$. Due to the intrinsic limitation of the 4D‐STEM approach at higher doses—particularly in regions with hole formation or complete amorphization—the ${\mathrm{PSNR}}^{-1}$ representation is only applicable where a sufficient degree of crystallinity remains. A more detailed discussion of this representation is provided in the “${\mathrm{PSNR}}^{-1}$ radial mapping and model deviations” section of the Supporting Information.

### 2.5 Radial Point–Spread and Long–Range Tail Behavior

**Figure 6:**
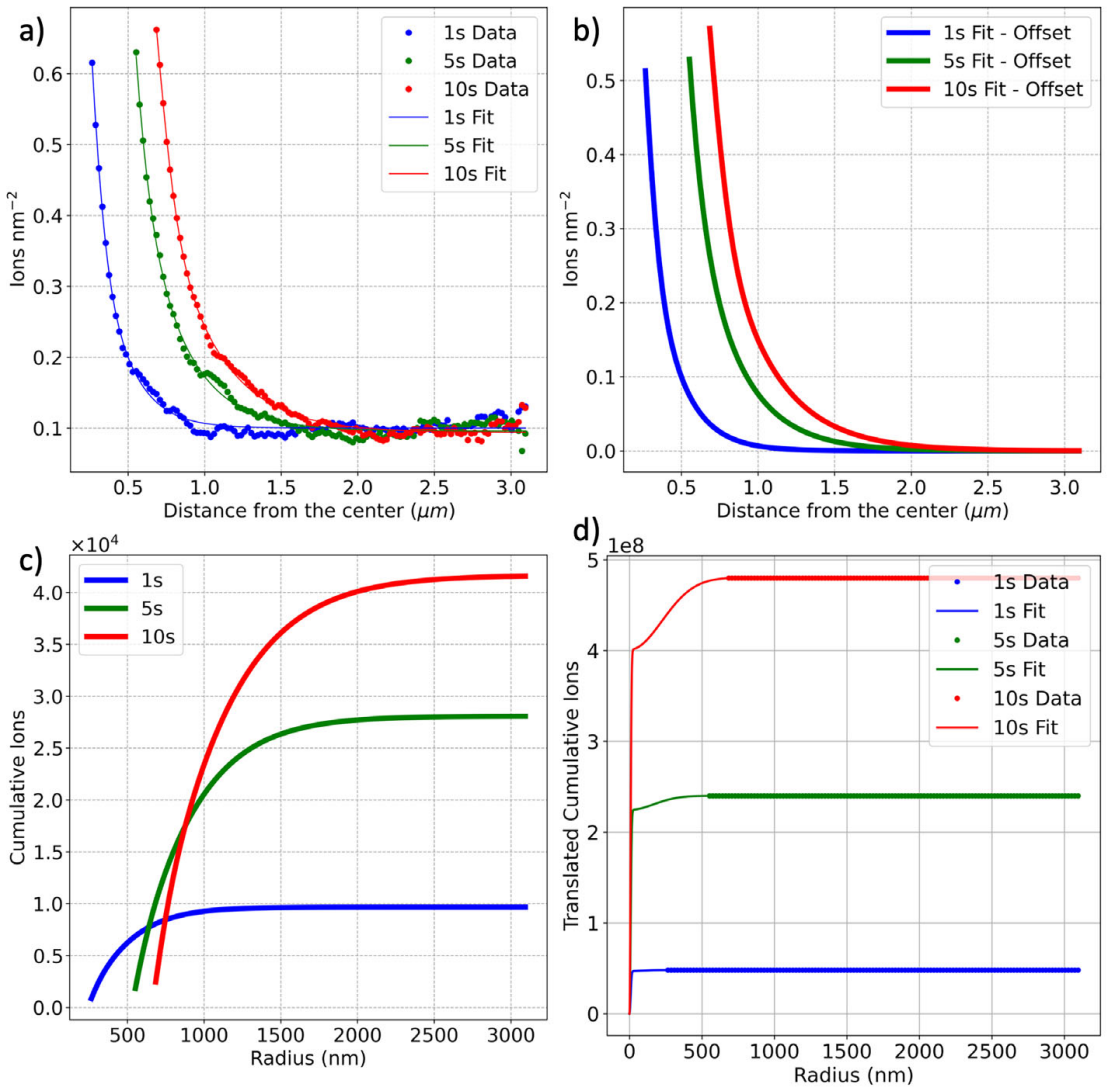
Procedure for obtaining the radial probability distribution of implantation events. a) The quantifiable ion density range from Figure 4 (in ions ${\mathrm{nm}}^{-2}$) was fitted using a combination of a Gaussian and an exponential function. b) Fitted ion density profiles. c) Fitted curves integrated to obtain the cumulative number of implanted ions as a function of radial distance. The non‐zero starting point reflects the exclusion of fully amorphous and hole regions, where the majority of ions are initially deposited. d) The far‐right end of the cumulative curves was offset to match the total expected number of implanted ions based on a 7.5 pA beam dwelled over times of 1 s (blue), 5 s (green), and 10 s (red). To fill the unquantified gap, a combination of two Gaussian components and an exponential tail was employed: the inner Gaussian was constrained to a fixed FWHM of ≈15 nm, representing the imaging resolution core, while the middle Gaussian—representing secondary scattering—was allowed to vary in FWHM during fitting.

Figure 6a plots the local ion dose (ions ${\mathrm{nm}}^{-2}$) as a function of radial distance from the beam center for three ${\mathrm{Ga}}^{+}$ dwell times (1 s, 5 s, and 10 s). These profiles were extracted from regions where the ion dose remained below the amorphization threshold, enabling reliable quantitative analysis. To capture the full radial damage distribution, Figure 6b shows fitted models comprising a broad Gaussian combined with an exponential tail. Figure 6c presents the cumulative ion dose as a function of radius, calculated from the fitted profiles. Because the saturated central region was excluded from the original dose measurements, these curves do not start at high values and underestimate the total ion dose near the beam center. To address the missing contribution from the saturated core, Figure 6d shows the reconstructed cumulative ion curves obtained by fitting the radial dose profiles with a composite model consisting of two Gaussian functions and an exponential tail (see Supporting Information for the mathematical formalism). The first Gaussian represents the tightly focused beam core and was fixed with a full width at half maximum (FWHM) of approximately 15 nm across all dwell times, consistent with the measured “imaging resolution.” The second Gaussian captures the broader mid‐range distribution and is allowed to vary in width to account for dose‐dependent broadening. The exponential component models the long‐range tail and defect‐mediated diffusion. The total integrated ion dose from the fits was constrained to match the known number of ions delivered for each dwell time, based on the beam current (7.5 pA) and exposure durations. This approach ensures that the reconstructed cumulative curves begin as zero at the beam center and accurately capture the full spatial distribution of ions.

**Figure 7:**
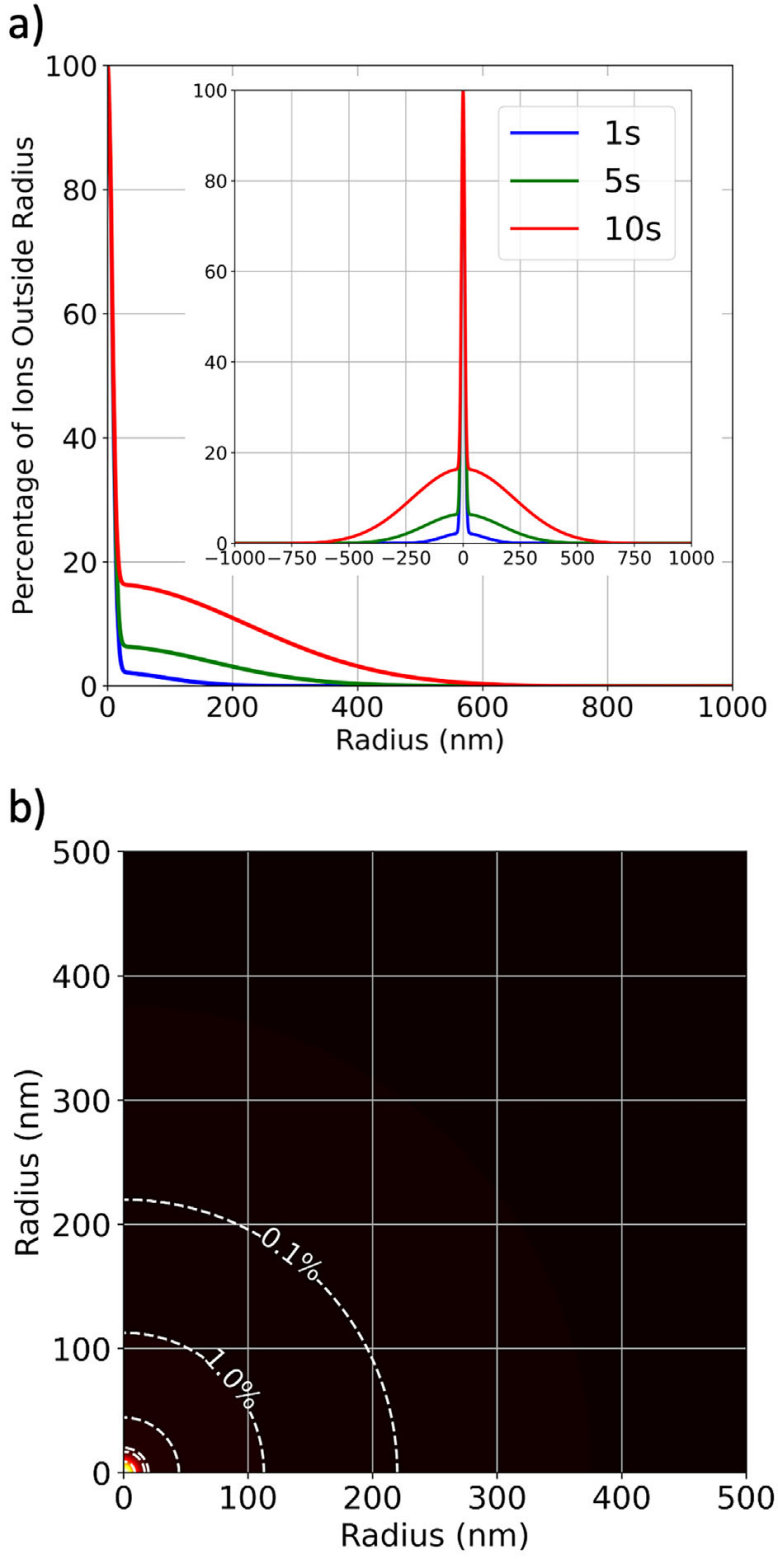
Radial probability contour plots derived from the integrated area under the fitted curves in Figure 6, combined with the known ion dose delivered during irradiation. (a) Displays a plot of percentage of ions outside a given radius. The inset is a vertically‐mirrored plot to illustrate the expected damage profile around and across a FIB‐dwelled spot. Panel (b) shows a two‐dimensional heat map representing the probability of finding ions beyond a given radius for the 1 s dwell time spot (total incident ions equals to $4.68\times 10^{7}$). The probability values are indicated by the dashed white contours over the plot.

Figure 7 translates the reconstructed dose profiles into radial probability distributions, showing the percentage of ions expected to land beyond a given radius. The mirrored plot in the inset of Figure 7a clearly shows that a FIB damage profile is a convolution of three different components, as expected from theoretical calculations [12]. The radial probability plots confirm that the core remains spatially confined, while the scattering tail increasingly dominates the long‐range distribution at extended distances. Figure 7b presents a two‐dimensional heat map illustrating the probability distribution of ion damage events as a function of radial distance for the spot dwelled for 1 s. This distinction has important implications for different FIB applications. In deterministic single‐ion implantation, the goal is to place exactly one ion per site with nanometer‐scale precision. Extrapolating from the measured distributions, the radial spread for a single‐ion event is expected to be extremely narrow—on the order of a few nanometers—due to the absence of cumulative scattering effects. This makes crosstalk negligible in arrays with pitches as small as 20–50 nm. In contrast, when using the focused ion beam for imaging fiducial marks prior to implantation, the dwell times often fall within the 1–10 s range studied here. Under these conditions, the spread of ions becomes more significant, and the scattering tails can extend far beyond the core region. In these scenarios, precision and careful planning become critical to avoid unintentional damage to nearby structures. This highlights the importance of being able to map the point spread function of the beam and tailor beam parameters to the spatial constraints of the device. In contrast, for higher dose applications such as lamella preparation and circuit repairs, the lateral extent of the amorphization layer is a critical parameter. The exponential tail in the ion distribution contributes to damage well beyond the beam center and means to mitigate the lateral damage footprint must be incorporated into the process.

The Supporting Information demonstrates the robustness of combining 4D‐STEM with PSNR analysis by significantly varying beam and camera acquisition parameters, while still achieving ultra‐sensitive defect detection and potential spatial resolution of few angstroms. Additionally, it explores the potential of electron backscatter diffraction (EBSD) as an alternative approach for achieving comparable fidelity in FIB profiling.

## 3 Conclusion

The primary objective of establishing the relationship between PSNR and ion dose is to enable reproducible, quantitative mapping of damage around localized ion beam exposures—particularly in regions where conventional imaging techniques fall short. We demonstrate how to convert PSNR values obtained from regions surrounding sites where the FIB was held stationary into spatially‐resolved ion dose estimates. PSNR metrics require a robust dataset with spread‐out pixels intensity that are intrinsically related to the crystal periodicity; which is met by using 4D‐STEM hypercubic datasets of CBED patterns. The approach allows to reconstruct the 2D distribution of implanted ions across and around the core impact region. Notably, the peripheral zone, which is often overlooked in ion beam damage studies, due to the lack of techniques that combine high sensitivity with nanoscale spatial resolution. Our method addresses a critical gap in knowledge by offering a new pathway for characterizing subtle damage gradients in ion‐irradiated materials, thereby enabling direct comparisons with implantation models and establishing a foundation for correlating structural damage with functional degradation in nanoscale devices.

## 4 Experimental Section

### 4.1 Silicon Membrane

The 30 nm thick single‐crystal Si membranes were commercially manufactured by Silson Ltd. The window edges and diagonals are oriented along the <110> and <100> directions, respectively, with (001) surface plane. Details on how Si membranes can be produced can be found in Refs. [35, 36, 37].

### 4.2 FIB Implantation Strategy

Singly charged gallium ions ($Ga^{+}$) with an energy of 30 keV were implanted using a dual‐beam focused ion beam microscope and scanning electron microscope (FIB‐SEM, FEI Nova Nanolab). The gallium ion current was measured before and after implantation using a Faraday cup and determined to be 7.5 pA on both readings and, therefore, was assumed constant throughout the implantation with an uncertainty of less than ∼1.5%. The conventional resolution test was performed by imaging a gold on carbon sample, capturing an image of 1024×1024 pixels^2^ with a dwell time of 30μs. For dark‐field scanning transmission electron microscopy (STEM) imaging, a line of milled spots in a 30 nm thick Si membrane was created by scanning the ion beam with a pitch of 5000 nm and dwell times exponentially increasing from 0.08 ms to 500 ms. For the 4D‐STEM measurements, the patterns consisted of two main components: the calibration squares and the milled spots. The 28 calibration squares had dimensions equal to 2500×2500 ${\mathrm{nm}}^{2}$ with a center‐to‐center spacing of 3000 nm. The ion dose in each square was determined by the dwell time for each scanned pixel (with a pitch size of 10.7 nm), varying exponentially (dose $=1.182\times 1,176^{j}$, being j the index from 1 to 26) from $1.39\;\mu C/\;cm^{-2}$ (repeated for three squares) to $80\;\mu C{\mathrm{cm}}^{-2}$. A $10^{-6}\;\times \;\left({\text{cm}}^{-2}/\left({\left(10^{7}\right)}^{2}\;{\text{nm}}^{2}\right)\right)\;\times \;\left(1/\left(1.6\;\times \;10^{-19}\;C\right)\right)$ conversion results in doses ranging from 0.09 ions ${\mathrm{nm}}^{-2}$ to 5.00 ions ${\mathrm{nm}}^{-2}$. For the milled spots in the beam profiling experiment, the beam dwelled a fixed pixel for 1 s, 5 s, and 10 s. Additional implantation was carried out to generate alignment marks, L‐shaped and triangular, with doses of $80\;\mu C{\mathrm{cm}}^{-2}$ and $1600\;\mu C{\mathrm{cm}}^{-2}$, respectively, as well as to flatten the membrane and mitigate out‐of‐plane buckling as suggested in [5, 8]. The sample was not annealed, i.e., no attempt was made to activate the Ga atoms and any damage due to the implantation was not repaired.

### 4.3 ADF–STEM Imaging Conditions

Annular dark‐field (ADF) images were acquired using a Thermo Scientific Talos F200i operated in STEM mode at 200keV, at “spot‐size” 5, with a selected intermediate condenser aperture size of 70μm, resulting in a convergence semi‐angle of around 10.5 mrad. The resulting screen current was ∼ 200pA and the 2048×512 pixels^2^ images were obtained using a 10μs dwell time. The holes and damage diameters were measured using the intermodes threshold method in ImageJ. The ADF‐STEM images related to the 4D‐STEM experiments were acquired in the Tescan TENSOR.

### 4.4 4D–STEM Data Acquisition

Diffraction datasets were acquired using a Tescan TENSOR 4D‐STEM microscope operated at an accelerating voltage of 100 keV. During scanning, the electron beam was dynamically precessed, and diffraction patterns (DPs) were recorded using a DECTRIS Quadro hybrid‐pixel direct electron detector. Two experimental configurations were employed to optimise data quality and spatial resolution. In the first configuration, the beam was precessed at 0.8 degrees, with a convergence semi‐angle of 12 mrad and a beam current of 100 pA. Under these conditions, diffraction patterns were acquired with a dwell time of 1 ms per scan point. In the second configuration, the beam was precessed at 0.7 degrees, with a convergence semi‐angle of 8 mrad and a beam current of 210 pA. A longer dwell time of 10 ms was used to enhance signal acquisition.

### 4.5 EBSD Data Acquisition

Kikuchi patterns in electron backscatter diffraction (EBSD) were acquired using a JEOL JSM‐7100F field emission scanning electron microscope (SEM) equipped with a Thermo Fisher Lumis EBSD detector. The region of interest was scanned with a 20 keV electron beam at a current of 0.95 nA, using a dwell time of 50 ms and a step size of 150 nm.

### 4.6 PSNR Computation Workflow

**Figure 8:**
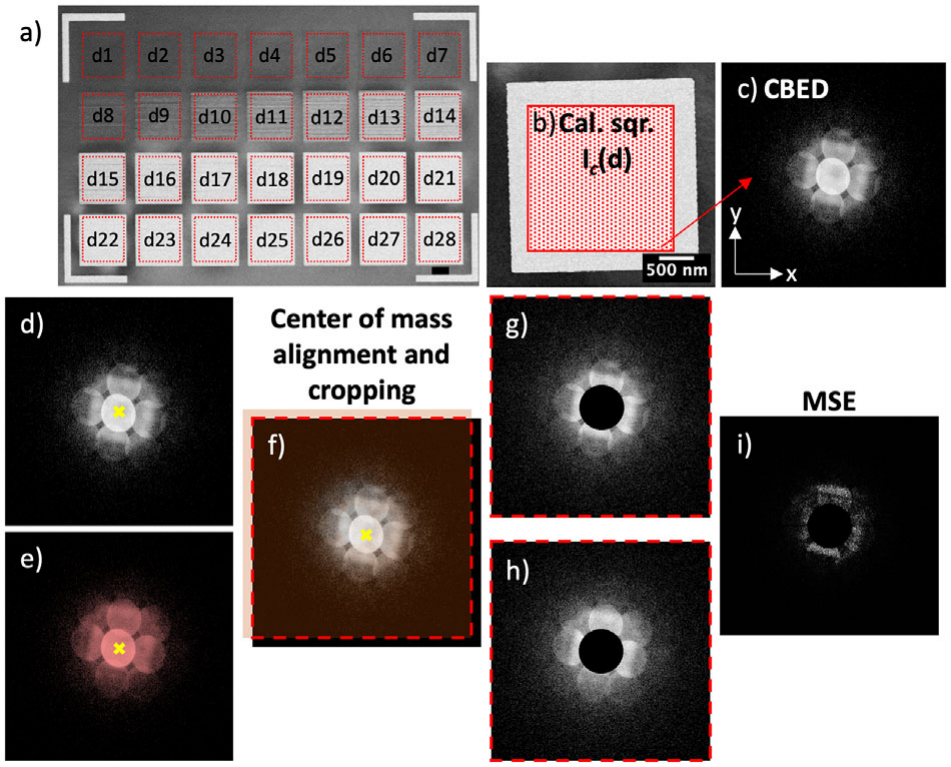
Workflow for quantifying damage in single‐crystal silicon using convergent beam electron diffraction (CBED) and peak signal‐to‐noise ratio (PSNR) metrics. (a) Scanning electron microscopy (SEM) secondary electrons image of a 30 nm‐thick single‐crystal Si membrane implanted with 30 keV ${\mathrm{Ga}}^{+}$ ions. Dashed‐line rectangles correspond to scanned regions inside areas implanted with a known ion dosage increasing from d1 = 0.09 ions ${\mathrm{nm}}^{-2}$ to d28 = 5.00 ions ${\mathrm{nm}}^{-2}$. b) High‐magnification annular dark‐field STEM (ADF‐STEM) image of one calibration square, illustrating the 100×100 CBED patterns (DPs) acquired over a $4\;\mu m^{2}$ area. c) Example CBED pattern from this dataset. d,e) Representative DPs from a reference (unimplanted) region just above the squares in (a) and from one of the implanted regions, respectively. Yellow crosses mark the center‐of‐mass in each DP. f) Overlay of the reference and implanted DPs after aligning their center‐of‐mass. Pixels outside the red dashed square are cropped to standardise the array shape, generating equally sized arrays like (g) and (h), prior to mean squared error (MSE, in (i)) and PSNR calculations. In panels (g–i), the black ellipses mark the masks employed to exclude contributions from the zero–order discs, thereby minimizing the effects of thickness–related intensity variations.

Using dynamic precession electron diffraction, a quasi‐kinematical dataset comprising a broad range of measured reflections was acquired at each dwell point. The implanted squares highlighted by red dashed squares in Figure 8a served as calibration regions, $I_{c}\left(d\right)$. A total of n=10000 pixels were scanned over a 2μm × 2μm area, deliberately avoiding the edges (Figure 8b). Each dwell point produced a 512×512 pixel convergent beam electron diffraction (CBED) pattern (Figure 8c). The analysis was based on the peak signal‐to‐noise ratio (PSNR). For this, each CBED pattern from the implanted regions was compared to 10 randomly selected CBED patterns (from the 10 000 acquired) in a pristine area adjacent to the calibration squares region in Figure 8a. In total, 2 800 000 comparisons were performed—100 000 for each of the 28 implanted squares with ion dose d in ions per nm.

The pixel‐wise standard deviation across all 10 000 reference patterns was calculated as:

(4) $$\sigma_{r}\left(x,y\right)=\sqrt{\frac{1}{n}\sum_{j=1}^{n}{\left(I_{r}^{\left(j\right)}\left(x,y\right)-\bar{I_{r}}\left(x,y\right)\right)}^{2}}$$

where $I_{r}^{\left(j\right)}\left(x,y\right)$ is the intensity at pixel (x,y) in the $j^{\text{th}}$ diffraction pattern, and $\bar{I_{r}}\left(x,y\right)$ is the mean intensity at that pixel across all *n* = 10 000 reference patterns. The maximum observed standard deviation was approximately 8 intensity units. Given the 16‐bit grayscale range of the CBED patterns (MAX*_I_* = 2^16^ − 1 = 65 535), this corresponds to a relative variation of less than 0.013% of the expected full intensity range, indicating low variability. Therefore, a subset of 10 reference patterns was deemed sufficient for PSNR calculations without compromising accuracy while maintaining realistic processing times.

For each comparison between reference and target patterns, the center of mass was calculated, Figure 8d,e, and aligned via offsetting. Excess edges were trimmed, outside of dashed lines in Figure 8f, to ensure alignment, and the resulting dimensions of each trimmed target/reference pair were recorded. At the end of the loop, all pairs were resized to match the smallest dimensions, M×N, by trimming random edges, Figure 8g,h, ensuring uniformity across the dataset. The zero‐order disks were masked to minimize the influence of membrane thickness variations from sputtering, ensuring that the PSNR analysis primarily reflects beam‐induced damage.

The mean squared error (MSE) between each calibration pattern $I_{c}^{\left(i\right)}\left(x,y\right)$ and 10 random pixels from the reference region $I_{r,\text{rand}}\left(x,y\right)$ was calculated as

(5) $$\mathrm{MSE}{\left(d\right)}^{\left(i\right)}=\frac{1}{MN}\sum_{x=1}^{M}\sum_{y=1}^{N}{\left(I_{c}{\left(d\right)}^{\left(i\right)}\left(x,y\right)-I_{r,\text{rand}}\left(x,y\right)\right)}^{2}$$

The peak signal‐to‐noise ratio (PSNR) was then calculated as:

(6) $$\mathrm{PSNR}{\left(d\right)}^{\left(i\right)}=10\cdot \log_{10}\left(\frac{{\left({\mathrm{MAX}}_{I}{\left(d\right)}^{\left(i\right)}\right)}^{2}}{\mathrm{MSE}{\left(d\right)}^{\left(i\right)}}\right)$$

for each of the 10 MSE values for each pixel and dose, where ${\mathrm{MAX}}_{I}{\left(d\right)}^{\left(i\right)}$ is the maximum bit intensity measured in $I_{c}^{\left(i\right)}\left(x,y\right)$ for the $i^{\text{th}}$ pixel in the calibration square $I_{c}\left(d\right)$ from i=1 to i=n=100000. PSNR is expressed in decibels (dB), with higher values indicating greater similarity (i.e., lower noise or distortion). In this study, PSNR values across the calibration dataset ranged from approximately 15–30 dB. While PSNR values for 16‐bit natural images typically range from 60 to 80 dB in image compression literature [38], the values observed here reflect the fundamentally different nature of the data.

## Conflicts of Interest

The authors declare no conflicts of interest.

## Supporting information


**Supporting File**: smtd70517‐sup‐0001‐SuppMat.pdf.

## Data Availability

The data that support the findings of this study are available from the corresponding author upon reasonable request.
