# Supplementary material for: Quantifying the Full Damage Profile of Focused Ion Beams via 4D‐STEM Precession Electron Diffraction and PSNR Metrics
Source: Small Methods. 2026 Feb 12;10(5):e02258. doi: 10.1002/smtd.202502258 (PMC12972287; doi:10.1002/smtd.202502258)
Supplement: Supplementary file 1 — Supporting File: smtd70517‐sup‐0001‐SuppMat.pdf. [file SMTD-10-e02258-s001.pdf]

SUPPORTING INFORMATION: Quantifying the Full Damage Profile of Focused Ion Beams via 4D-STEM Precession Electron Diffraction and PSNR Metrics

M.G. Masteghin[1,2,*], Z.P. Aslam[3], A.P. Brown[3], M.J. Whiting[4], S.K. Clowes[1], R.P. Webb[5], D.C. Cox[1,5,6]

[1] Advanced Technology Institute, University of Surrey, Guildford, GU2 7XH, UK
[2] DTU Nanolab, Technical University of Denmark, Fysikvej, Kongens Lyngby 2800, Denmark
[3] School of Chemical and Process Engineering, University of Leeds, Leeds, LS2 9JT, UK
[4] School of Mechanical Engineering Sciences, University of Surrey, Guildford, GU2 7XH, UK
[5] Ion Beam Centre, University of Surrey, Guildford GU2 7XH, UK
[6] National Physical Laboratory, Teddington, TW11 0LW, UK
[*] Corresponding author: matmas@dtu.dk

CORRECTION OF ADF-STEM OFF-AXIS ARTEFACTS

We designed an experiment in which twenty-eight 2.5×2.5 µm^2 squares were implanted with a 30 keV Ga^+ beam at doses ranging from 0.09 ions nm^{-2} to 5 ions nm^{-2} **(Figure S1a)**. By imaging these squares using low-angle annular dark-field (LAADF-STEM), we can produce a calibration curve of grayscale levels as a function of ion dose (Figure S1b), which will be correlated with a scan around ion-milled holes. It is important to note that when imaging in STEM mode using LAADF detection, the events are detected just outside the bright-field cone, collecting electrons that have been coherently scattered at low-angles (e.g. slightly damaged regions) or diffuse scattering from the near-forward electrons through the amorphous regions (i.e., reduced channelling) [1].

In Figure S1c, the row of holes on the right of the triangles (fiducial marks) was dwelled at 1 ms, 5 ms, and 10 ms from top to bottom, respectively, with the insets' region highlighted in red indicating where a hole is located. During LAADF-STEM imaging of the implanted squares, we observed a systematic grayscale offset across the scan area. This artifact arises from optical off-axis effects, which persist even when operating in descan mode with a two-stage deflection coil. The artifact manifests as a gradient in image intensity, causing squares with identical ion doses to appear with different grayscale levels, as highlighted by the offset between the orange and green curves in Figure S1b. The origin of this effect is attributed to imperfect beam steering and minor misalignments of the optical axis relative to the crystal zone axis and, finally, to the detector collection angle. At large collection angles, ADF contrast is dominated by Rutherford-like scattering proportional to $Z^{1.7-2}$; whereas at low-angles diffraction, the contrast transfer function oscillations with defocus and aberrations become increasingly important. Any residual optical offset leads to spatial variations in scattering angle and collection efficiency, thus producing a continuous intensity gradient. To mitigate these artifacts, all calibration squares and regions of interest must be acquired within a single field of view — and as small as possible. Additionally, post-acquisition processing such as background subtraction or normalization using reference regions can reduce the effect of the offset gradient. However, these methods cannot fully account for local variations in contrast transfer. For this reason, we proposed a 4D-STEM experiment in which a full diffraction pattern is recorded at every probe position. Such a dataset would enable center-of-mass correction and virtual detectors post-processing, providing a more robust and quantitative measurement of ion-induced damage.

PSNR^{-1} RADIAL MAPPING AND MODEL DEVIATIONS

The shape of the PSNR^{-1} curves (**Figure S2**) in our data resembles the damage profiles reported in Figure 6 of Drezner et al. [2], which were modelled using a combination of two Gaussian functions and an exponential decay:

$$D(r) = A_1 e^{-\frac{r^2}{2\sigma_1^2}} + A_2 e^{-\frac{r^2}{2\sigma_2^2}} + A_3 e^{-\lambda r} + C, \qquad \text{(S1)}$$

where D(r) represents the damage intensity — represented here as the inverse of the PSNR, based on the established correlation that lower PSNR values correspond to higher degrees of amorphization — as a function of radial distance (r), with A_1, A_2, and A_3 as amplitude coefficients, σ_1 and σ_2, as the standard deviations of the two Gaussian components, λ as the decay constant of the exponential tail, and C as a constant to correct for the non-zero ion density at regions far from the implantation spot. In this composite model, the narrow Gaussian

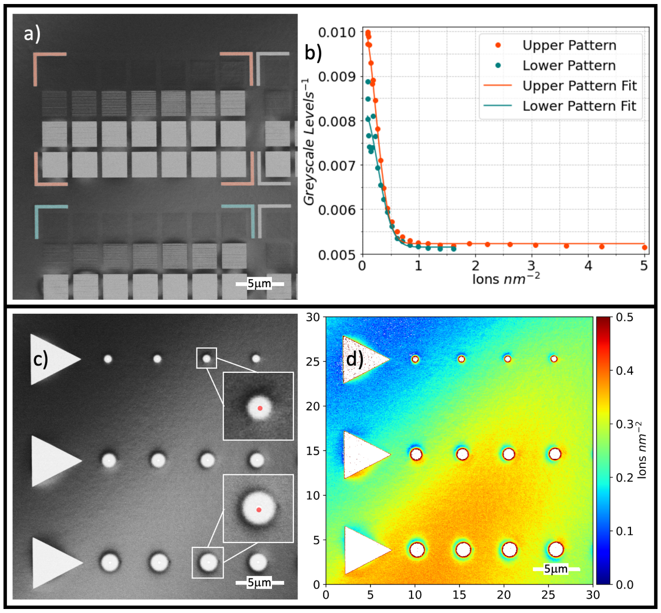

Figure S1. Tentative of damage quantification using LAADF-STEM signal. a) Calibration 2.5 × 2.5µm^2 squares created by 30 keV Ga$^+$ implantation with doses exponentially ranging from 0.09 ions nm$^-$2 (repeated three times) to 5 ions nm^{-2} (from darker to lighter tones as explained previously), contained within the fiducial marks highlighted in either orange or green. b) One-over-grayscale levels plotted as a function of the calibration doses. Levels were obtained by averaging the intensity of individual pixels over a 4 μm^2 area within each square. c) LAADF-STEM image obtained at 100 keV for three sets of dwelled holes (1 ms, 5 ms, and 10 ms from top to bottom. Insets highlight the holes' area in red, surrounded by damaged silicon. d) 2D heat map of expected dose as a function of spatial position obtained by cross correlating the pixels' bit intensities in (c) to the orange calibration curve in (b). Regions marked in white correspond to doses exceeding 5 ions nm^{-2}.

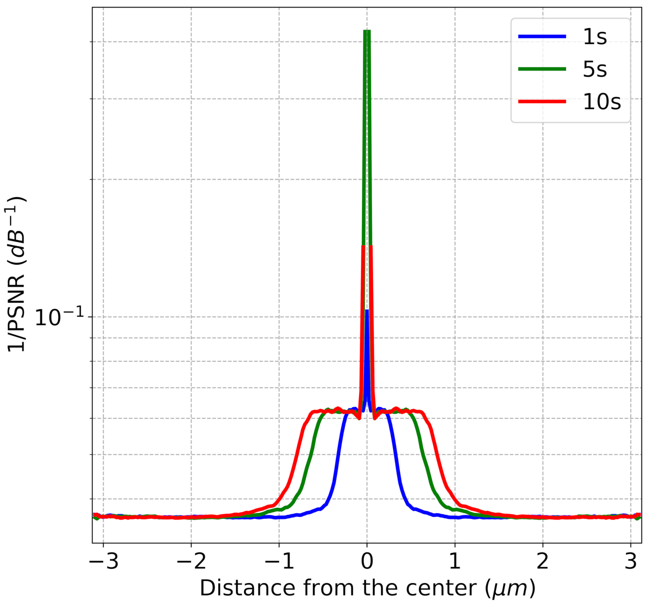

Figure S2. Point spread function (PSF) preliminary profiles obtained by applying two successive mirroring operations to reconstruct a full circle from a single quadrant (shown in Figure 5d-f), enabling the extraction of radial intensity profiles represented in terms of 1 over PSNR in logarithmic scale. The resulting curves were interpreted as a combination of two Gaussian functions and an exponential tail, with the Gaussians saturation attributed to the limitations of the PSNR method in quantifying degrees of amorphousness after crystal symmetry is lost.

reflects the high-dose core focused along the FIB optical axis, effectively corresponding to the imaging resolution. The broader Gaussian accounts for non-focused neutral ions, intermediate-range scattering and the spatial spread of ion-induced cascades — including subsurface straggling, low-angle recoil trajectories, and lateral channelling effects —; while the exponential term should account for long-range scattering at low-vacuum regimes or radiation-induced diffusion processes.

Despite the qualitative agreement with the Drezner et al. [2] model, we observe deviations in the peak intensity and relative width of the fitted components. Notably, the amplitude of the narrow Gaussian appears reduced, and the broader Gaussian flattens toward the centre. This discrepancy occurs from PSNR saturation in regions where the local dose exceeds 0.8 ions nm^{-2}, beyond which Bragg contrast is fully lost, and additional damage becomes indistinguishable via 4D-STEM. As a result, damage is systematically underestimated in the high-dose core. Importantly, we also find that the full width at half maximum (FWHM) of the damage profiles does not scale linearly with dwell time. For example, the FWHM of the 1 s profile is not one-fifth or one-tenth the size of those corresponding to 5 s or 10 s exposures. This non-linear scaling highlights fundamental characteristics of focused ion beam interactions: while the number of delivered ions increases linearly with dwell time, the lateral extent of damage broadens sub linearly due to several factors. First, ion–solid interactions are probabilistic and scattering events are governed by fixed angular distributions, meaning that the lateral range of displaced atoms is bounded. Second, at higher doses, collision cascades begin to overlap, and newly displaced atoms may recombine with existing vacancies or become trapped in pre-damaged regions, limiting further lateral propagation. Third, amorphization itself modifies the material structure, potentially reducing channelling efficiency and changing ion penetration and scattering dynamics. Finally, cumulative thermal effects and radiation-enhanced diffusion may contribute to broader profiles at longer dwell times, but only weakly and non-linearly. Altogether, these mechanisms should lead to a saturation in damage width, consistent with prior findings in TRIM [3] simulations, and better illustrated in **Figure S3**. The

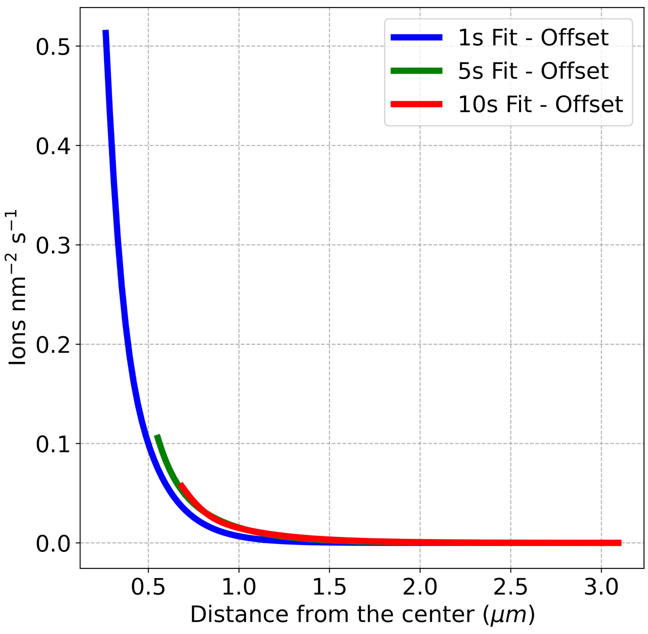

Figure S3. Time-normalized ion density profiles. Each curve was obtained by fitting a broad Gaussian combined with an exponential decay to the quantifiable regime (prior to amorphization or through-thickness milling) around the FIB-dwelled spots, followed by normalization to the respective dwell time.

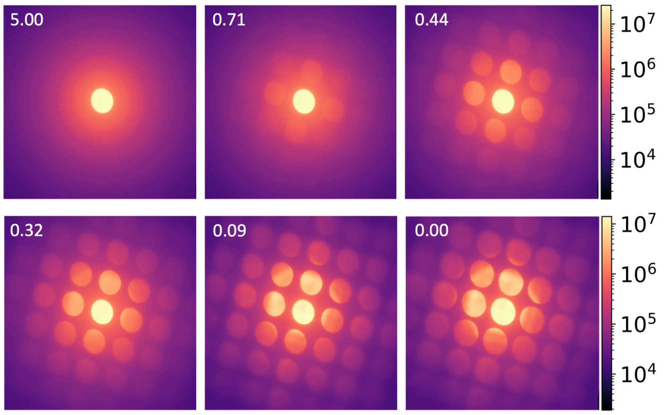

Figure S4. 10,000 integrated CBED patterns obtained by scanning the 0.7 degrees precessed 100 keV, 8 mrad convergence semi-angle, 210 pA probe current with a 10 ms dwell time per pixel. The CBED patterns have dimensions of 512×512 pixels and are plotted using a logarithmic intensity scale. The numbers at the top-left of each CBED pattern indicate the dose (in ions nm^{-2}) used in the corresponding calibration square, with 0.00 representing the pristine region.

plots in Figure S3 were obtained by normalising the fits from Figure 6b by the respective dwell times (i.e., dividing the ion density by 1, 5, and 10 for increasing dwell times). Although uncertainties in amplitude may still influence the probability plots in Figure 7, the closer proximity of tail widths for the 5 s and 10 s curves suggests that the long-range damage distribution approaches saturation with increasing dwell times. These assumptions can be further supported through complementary modelling (e.g., Monte Carlo [4, 5] or TRIDYN [6, 7]).

CONVERGENCE SEMI-ANGLE TRADE-OFF EXPERIMENTS

To investigate the impact of probe parameters on 4D-STEM/PSNR output while mapping FIB PSFs, the convergence angle was reduced from 12 mrad to 8 mrad. A smaller convergence angle produces a larger real-space probe that could be a potential limitation, but in most 4D-STEM applications the scanning pitch size is larger than the probe diameter. Simultaneously, the reduced angular spread in reciprocal space results in sharper and more spatially confined CBED disks. This narrowing of the diffraction disks enhances the precision of center-of-mass measurements, which in turn should improve the technique sensitivity to subtle lattice distortions and strain fields associated with defects. Moreover, the reduced CBED size minimizes overlap between the central beam and higher-order diffraction features, lowering background interference and improving the interpretability of local intensity variations. These factors should collectively improve the accuracy and sensitivity of defect quantification in materials. Representative integrated CBED patterns acquired at lower convergence angles at same regions of those in Figure 4 are presented in **Figure S4**.

Figure S5 demonstrates the application of 4D-STEM with a higher convergence angle micro-probe to quantitatively map ion implantation dose around FIB-milled holes. In Figure S5(a), a calibration curve is established by comparing PSNR values from CBED patterns acquired in regions of known ion implantation dose to a reference region, as described previously. Figure S5(b-d) show ADF images of regions surrounding holes milled with Ga^+ beam dwell times of 1 s, 5 s, and 10 s, respectively. These serve as the spatial context for the subsequent PSNR and ion density mapping. For each of these regions, a 4D-STEM scan was performed, and the CBED pattern at each pixel was compared to reference CBEDs obtained from the far-left periphery of the scanned area, assumed to be undamaged. The resulting PSNR maps are shown in Figure S5(e-g), where lower PSNR values (blue) indicate higher degrees of amorphization. To translate these PSNR values into ion dose, the PSNR of each pixel was correlated to the calibration curve in Figure S5(a). The resulting 2D ion density maps are shown in Figure S5h–j, corresponding to the 1 s, 5 s, and 10 s dwell times, respectively. Note that pixels marked in black correspond to the vacuum region (hole) or the highly damaged (amorphous) regions. By selecting a reference region closer to the area of interest — leveraging

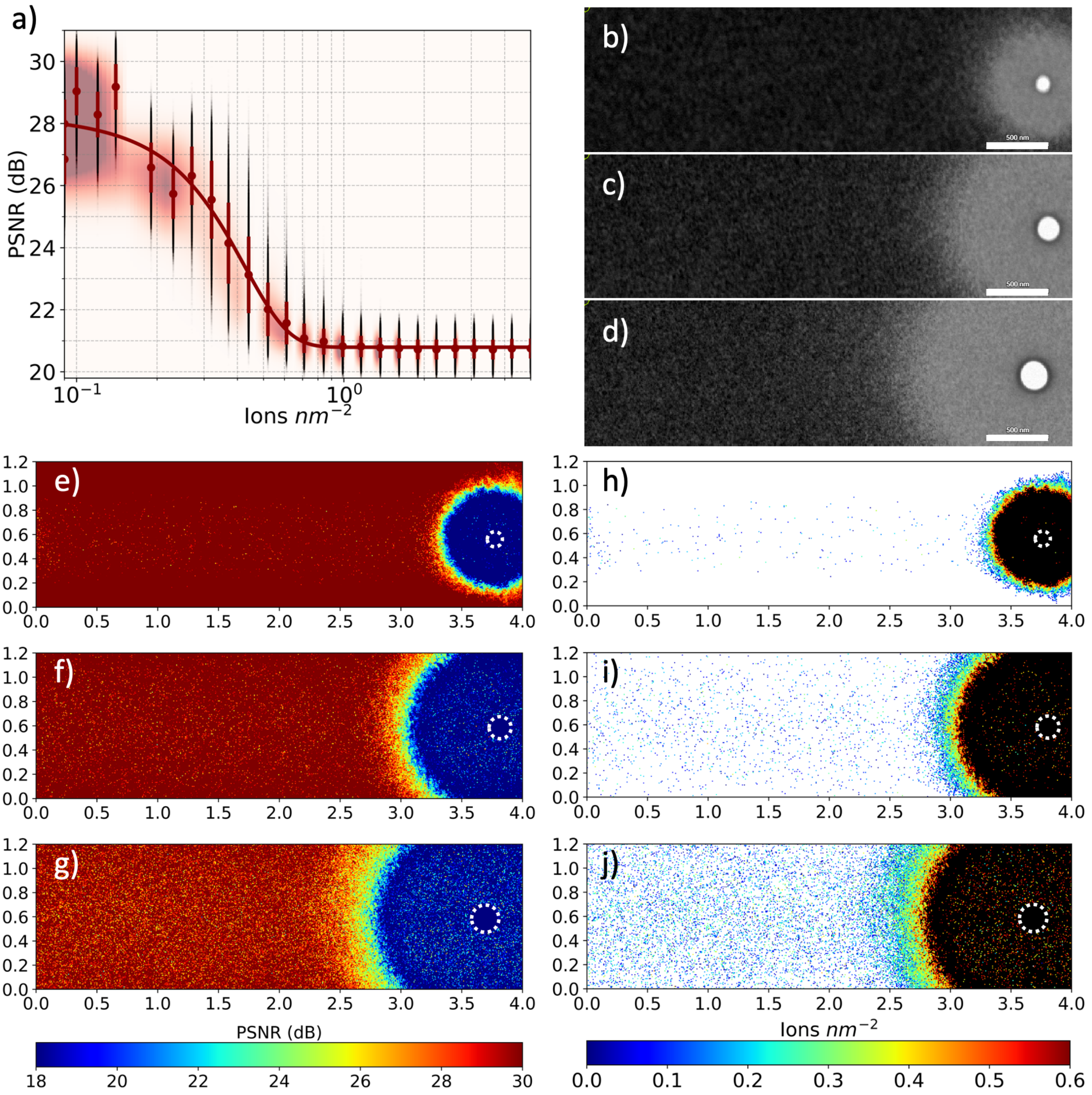

Figure S5. Overview of 4D-STEM and PSNR analysis for mapping the point spread function in focused ion beam-damaged regions. a) PSNR as a function of calibration ion dose for a new measurement set acquired with a smaller probe convergence semi-angle. Each black point represents one of 100,000 values collected per dose; red points indicate the median PSNR, with vertical bars denoting the error range. The red curve represents a Gaussian error function fit. b–d) ADF-STEM images of regions adjacent to holes milled using a 30 keV Ga^+ beam at 7.5 pA for 1 s, 5 s, and 10 s, respectively. e–g) 2D PSNR maps corresponding to the regions in (b–d), where each PSNR value was computed by comparing an individual CBED pattern to 10 randomly selected CBED patterns from a reference region, defined here as the far-left area just outside the field of views shown in b-d. Mean PSNR values were plotted. h–j) 2D ion density maps obtained by converting PSNR values to ion dose (in ions nm^{-2}) using parameters from the Gaussian error function fit. Regions marked with black dots represent areas below the lower PSNR plateau (i.e., heavily damaged), while white areas correspond to PSNR values indicative of doses below 0.09 ions nm^{-2} (i.e., pristine regions). Dashed white circles indicate the locations of the milled holes.

the beam point spread function (PSF) characterized in the previous experiment (see Figure 5) — we were able to correctly assign the region outside the beam core as defect-free without the need to add a constant to the fit of Equation (S1).

Further investigation is needed to determine the limit of detection across different convergence semi-angles. One possible approach would be to use implanted vertical lines with reducing ion concentration, combined with a scan perpendicular to the lines — as in the methodology reported in [8] for synchrotron X-ray nanoscopy. Notably, this experiment demonstrates the robustness of the method by testing a distinctly different probe configuration, varying the position of the reference region, and adjusting analysis parameters. It also accounts for a slight zone-axis misalignment consistent across all scans. These variations did not significantly affect the resulting ion density maps, in contrast to conventional ADF imaging, which is more prone to artifacts.

EBSD AS A POTENTIAL TOOL: OPPORTUNITIES AND LIMITATIONS TO BE EXPLORED

Finally, we note that electron backscatter diffraction (EBSD) can also be employed to investigate the point spread function of focused ion beams. EBSD can be performed directly on bulk samples, eliminating the need for electron-transparent lamella or membrane preparation, which is required for 4D-STEM. Additionally, the use of high beam currents in EBSD enables rapid data acquisition, provided that the sample can tolerate elevated electron doses and defects mobility under the electron beam is not a concern. Nevertheless, EBSD is inherently limited in spatial resolution compared to 4D-STEM. Its resolution, governed by the backscattered electron interaction volume (typically ≈20 nm in Si at 20 keV), is insufficient to resolve closely spaced or singular defects, even if the overall sensitivity to local lattice distortions is comparable — a question still under investigation. By contrast, 4D-STEM, with its sub-nanoscale probe and precision diffraction-based measurements, offers superior spatial resolution and localization capabilities, making it more suitable for quantifying fine-scale damage features. Figure S6 presents a representative PSNR map from EBSD measurements, acquired in a region comparable to that shown in Figure 4a. The diffuse edges observed around the implantation sites are attributed to electrostatic charging effects, likely due to sub-optimal grounding of the EBSD detector. Future studies will aim to systematically assess the detection limits of EBSD for defect quantification, particularly following a planned upgrade of the current system.

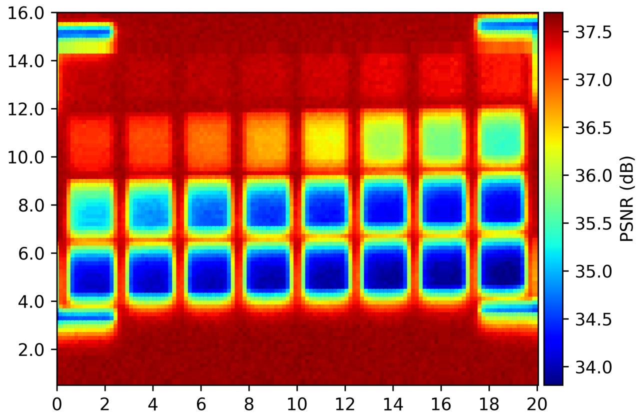

Figure S6. Electron backscatter diffraction (EBSD) data acquired from a region similar to that shown in Figure 4a. For simplicity, the PSNR analysis was performed without applying centre-of-mass alignment. Kikuchi patterns were compared to an averaged reference pattern obtained from a region located at the bottom of the imaged area. The diffuse edges observed in the maps are attributed to charging effects.

[1] Peter D Nellist. *The principles of STEM imaging*, pages 91–115. Springer, 2010.

[2] Yariv Drezner, Yuval Greenzweig, Shida Tan, Richard H Livengood, and Amir Raveh. High resolution tem analysis of focused ion beam amorphized regions in single crystal silicon—a complementary materials analysis of the teardrop method. *Journal of Vacuum Science & Technology B*, 35, 2017.

[3] James F Ziegler and Jochen P Biersack. *The stopping and range of ions in matter*, pages 93–129. Springer, 1985.

[4] E Morvan, Ph Godignon, J Montserrat, J Fernández, D Flores, J Millán, and J P Chante. Montecarlo simulation of ion implantation into sic-6h single crystal including channeling effect. *Materials Science and Engineering: B*, 46:218–222, 1997.

[5] A Hossinger. Accurate three-dimensional simulation of damage caused by ion implantation. In *Proc. 2nd Int. Conf. on Modeling and Simulation of Microsystems*, pages 363–366, 1999.

[6] W Möller and W Eckstein. Tridyn—a trim simulation code including dynamic composition changes. *Nuclear Instruments and Methods in Physics Research Section B: Beam Interactions with Materials and Atoms*, 2:814–818, 1984.

[7] W Möller and W Eckstein. Ion mixing and recoil implantation simulations by means of tridyn. *Nuclear Instruments and Methods in Physics Research Section B: Beam Interactions with Materials and Atoms*, 7:645–649, 1985.

[8] Mateus G Masteghin, Toussaint Gervais, Steven K Clowes, David C Cox, Veronika Zelyk, Ajith Pattammattel, Yong S Chu, Nikola Kolev, Taylor J Z Stock, and Neil J Curson. Benchmarking of x-ray fluorescence microscopy with ion beam implanted samples showing detection sensitivity of hundreds of atoms. *Small Methods*, 8:2301610, 2024.
